# Epidemiological profile of exogenous poisoning in children and adolescents from a municipality in the state of Mato Grosso☆

**DOI:** 10.1016/j.rpped.2014.06.002

**Published:** 2014-12

**Authors:** Felipe Ferreira S. Oliveira, Eliane Aparecida Suchara

**Affiliations:** Universidade Federal de Mato Grosso (UFMT), Barra do Garças, MT, Brazil

**Keywords:** Poisoning, Adolescent, Child

## Abstract

**OBJECTIVE:**

To study the epidemiology of exogenous intoxications in children and adolescents of Barra Garças, Mato Grosso, from January 2008 to September 2013.

**METHOD:**

This was a cross-sectional, retrospective, and descriptive epidemiological study. Data were collected from the Disease Notification System (Sistema de Informação de Agravos de Notificação [SINAN]) of the municipality, processed using Microsoft Excel, and evaluated through BIOESTAT statistical software. The variables included were: sex; age; toxic agent; time and place of service; route of administration; circumstance; and classification of intoxication. The age range was established according to the Brazilian Institute of Geography and Statistics, comprising children aged from 0 to 9 years old and adolescents aged from 10 to 19 years old.

**RESULTS:**

A total of 125 cases of accidental exogenous poisoning was registered, including 77 children and 48 adolescents. Food and beverages (38.4%) and drugs (24.0%) were the most common groups of toxic agents responsible for the poisoning. The largest age group affected by intoxication was composed of children aged from 0 to 4 years old (43.2%) and adolescents aged from 10 to 14 years old (19.7%). Regarding the circumstances, intoxication occurred due to suicide attempts (16.8%) and accidental events (23.2%) in adolescents and children, respectively. The study revealed a higher frequency of poisoning in girls.

**CONCLUSION:**

Exogenous intoxications occurred predominantly in children up to 4 years old, through the accidental consumption of food or drinks. Thus, the adoption of educational prevention programs for children's family members and caregivers is necessary.

## Introduction

Poisoning is defined as a clinical manifestation of the adverse effects caused in a living organism as a result of its interaction with some chemical (exogenous) substance.^1^ Every year, thousands of cases of poisoning are recorded in Brazil, caused by the ingestion of contaminated food, medications, use of pesticides, household cleaning products, veterinary products, and other chemical substances.^2^

Poisonings, mainly the unintentional, constitute one of the main causes of emergency pediatric care.^3^ Regarding the main elements associated with risks of poisoning among children, it can be observed that they are related to oral exposure and medication consumption, most of which happens in the household itself, where the presence of parents does not prevent its occurrence.^4^^,^ 5 Moreover, these poisonings are more common in families with more than three children and parents with low educational level, and in low-income families.^6^

It is observed that accidental poisoning, typical of pediatric patients, decreases during the course of the child's emotional and cognitive development; however, it becomes a possible escape route for problems in adolescence, due to the maturation of the concept of death.^7^ Detailed studies are required for adolescents, with greater focus on the circumstances and the intention of these poisonings.^8^ Therefore, a correct history of these children and adolescents should be obtained, to establish the appropriate treatment of these patients; however, pediatricians and physicians should be aware of the peculiarities of toxic agents that cause poisoning, considering age and time.^9^

In Brazil, data on poisonings are available in the annual publications of the National System of Toxic-Pharmacological Information (Sistema Nacional de Informações Tóxico-Farmacológica [SINITOX]), which compiles information from 36 Poison Control Centers (PCCs) located in 19 states and the Federal District.^3^ SINITOX 2010 data reported 29,554 cases of poisoning in children aged 0-9 years and 13,087 cases of poisoning in adolescents aged 10-19 years, with a higher prevalence of medications as the toxic agent in the age group of 1-4 years.^10^

In the Midwest Region, in 2010, SINITOX reported 3,533 poisonings in children aged 0-9 years and 1,371 cases in adolescents aged 10-19 years, with medications representing the most prevalent poisoning agent, mainly in the age range of 1-4 years.^11^ In relation to gender, a greater number of cases occurred in the female gender, with medications as the main causative agent.^12^ As for the circumstance, individual accidents prevailed, with a total of 6,099 cases; the second was attempted suicide, with 2,166 cases.^13^

Despite the existence of epidemiological data on poisonings at the national and regional levels, the reality in small towns remains unknown. Therefore, considering the vulnerability of children to poisoning, the exposure of young individuals to different toxic substances, together with the fact that there is little information in the literature on the characterization of poisonings in this age group in the municipalities of the state of Mato Grosso, this article aimed to describe the profile of exogenous poisoning in children and adolescents in Barra do Garças, state of Mato Grosso (MT), Brazil, from January of 2008 to September of 2013.

## Methods

This was a cross-sectional, retrospective, and descriptive epidemiological study regarding exogenous poisoning in children and adolescents from Barra do Garças (MT), from 2008 to 2013. The municipality is located in the geographical center of Brazil, with latitude 15°53'24" S and longitude 52°15'24" W; located at the foot of the Serra Azul mountain, an arm of the Serra do Roncador, drained by the rivers Araguaia and Garças. According to the Brazilian Institute of Geography and Statistics (IBGE), it had an estimated population of 57,791 in 2013.

The identification and characterization of the data collected for the study were established according to the Information System for Notifiable Diseases (Sistema de Informação de Agravos de Notificação - SINAN), through a notification form for exogenous poisoning distributed by the Ministry of Health. All reported cases of exogenous poisoning were analyzed, regardless of whether the poisoning was suspected or confirmed. The study period included January of 2008 to September of 2013, using a total of 125 forms. The variables evaluated were gender, age, toxic agent, time and place of service, route of administration, condition, and classification of poisoning. Regarding the classification of the studied age groups, they were defined according to the IBGE: children 0-9 years, and adolescents 10 to 19 years. 

Initially, a descriptive analysis was performed using Microsoft Excel 2010^(r)^ software (Microsoft - Washington, United States) and later the data were evaluated using the statistical software BioStat 5.0.^14^ Normality of the data was verified by the Shapiro-Wilks test and, subsequently, the nonparametric Kruskal-Wallis and Kolmogorov-Smirnov tests were applied. The confidence level for all analyzes was set at 95%. As for the coefficient of incidence, it was calculated according to the total number of recorded cases of poisoning during the study period for the population of the same place and period and multiplied by 1,000. The population data used in the study were based on the estimated population calculated by IBGE.

In compliance with Resolution 466/2012 of the National Health Council, the researchers submitted the present research to the Research Ethics Committee of Universidade Federal de Mato Grosso, which was registered and approved under CAAE No. 24213113.3.0000.5587.

## Results

Table 1 shows the distribution of the relative frequency (%) of cases of poisoning in children aged 0-9 years and adolescents aged 10 to 19 years, in the period 2008-2013, in Barra do Garças, MT, Brazil. The results demonstrate the occurrence of 77 cases of poisoning in children and 48 in adolescents, and the coefficients of incidence during the study period ranged between 0.05 and 0.64 per 1,000 inhabitants for children and between 0.03 and 0.27 per 1,000 inhabitants for adolescents. 

**Table 1:**
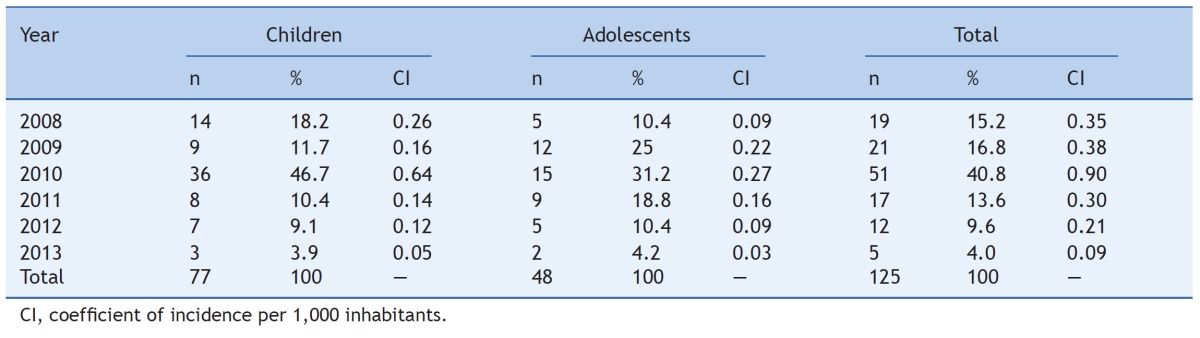
Distribution of poisoning cases in children and adolescents per year studied, in the municipality of Barra do Garças, MT.

The distribution of poisoning cases by age group in children and adolescents stratified by gender is shown in Table 2. It can be observed that >40% of all episodes of poisonings occurred in children aged 0-4 years. Regarding gender, in the age group of 0-14 years, there was a higher frequency of poisonings in male individuals. For the age group 15-19 years, female individuals were involved in most reported cases. There was a significant variation in the recorded number of poisonings when the number of poisoning cases was evaluated throughout the study years for the age group 0-4 years (*p*<0.05). 

**Table 2:**
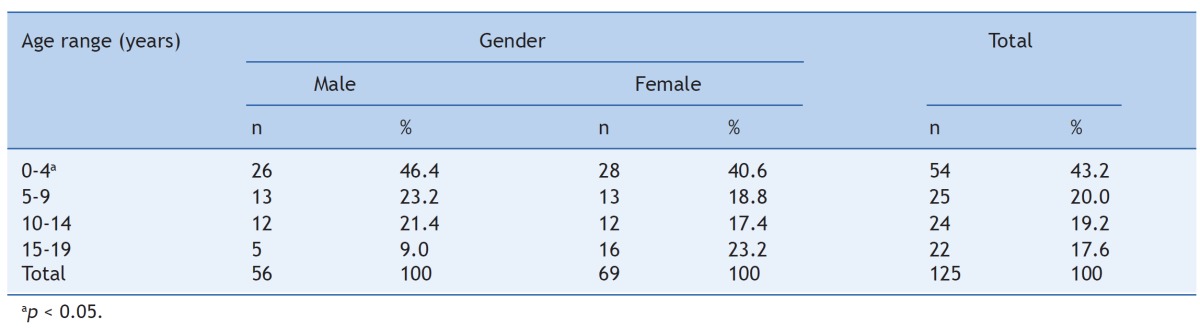
Distribution of poisoning cases by age group and gender.

Poisoning by age group according to the toxic agent, condition, route of administration, and classification are shown in Table 3. When assessing cases of poisoning by age group and toxic agent, the age group 0-4 years was the most prevalent, with 13 cases of drug poisoning and 16 cases through food or beverage. As for the toxic agent involved, food and drinks were predominant in the poisoning episodes in children and drugs in adolescents. It was also observed that among the different types of toxic agents found, there was a significant difference in the number of poisoning cases over the studied years (*p*<0.05). 

**Table 3:**
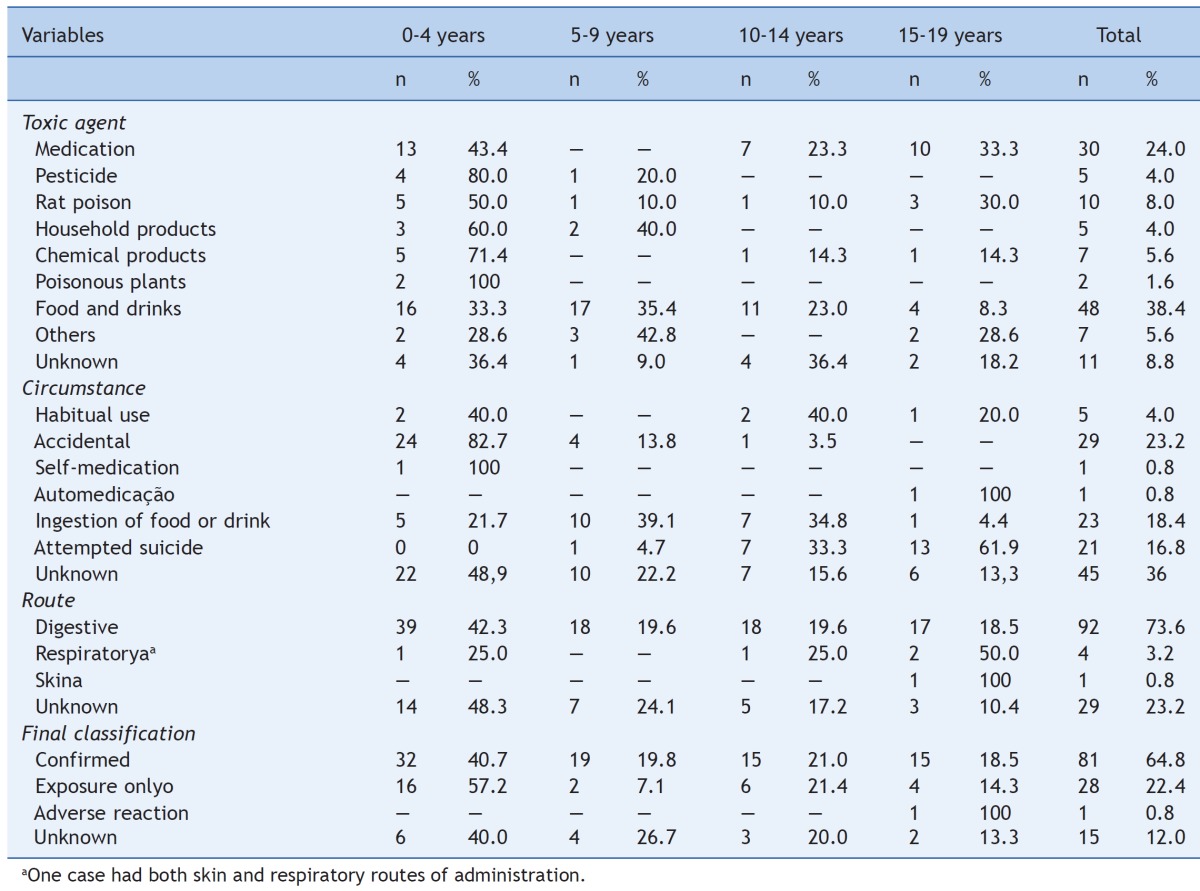
Classification of poisoning cases by age group (years), stratified by the toxic agent, circumstance, route of administration, and final classification.

As for the circumstance, the highest number of cases was due to accidental poisonings, with approximately 80% of these reported in children aged 0-4 years. Conversely, in cases of food or drink ingestion, approximately 60% of the poisonings affected the age group 5-14 years. When the number of reported poisonings recorded each year was assessed and compared with the reported circumstances of poisoning, there was a significant difference. Therefore, the number of cases of poisoning varied among the reported circumstances (*p*<0.05) over the years studied.

The route of administration with the highest number of poisonings was the gastrointestinal route, with 92 cases, and the treatment sites were the hospital (n=64) and outpatient clinic (n=49). Children aged 0-4 years were treated at the hospital and outpatient clinic in 23 (36%) and 26 (53%) cases, respectively. 

As for the time until treatment of the poisoning cases, children aged 0-4 and adolescents aged 15-19 totaled 36 poisoning cases treated within an hour. However, the reports showed a prevalence of notifications from which this information was missing. When assessing the monthly distribution of the number of poisonings, it was observed that the months of March, June, July, August, and November showed 22, 16, 20, 17, and 13 cases of poisoning, respectively. When assessing the final classification of the case, of the total of 125 reported cases, 81 were confirmed.

## Discussion

In the period studied, a higher incidence of poisoning was observed in children and adolescents in 2010; these numbers ​​were not related to any event of collective poisoning, as the cases had a constant distribution during all months of the year. Consequently, no outbreak of poisoning was identified. It was also observed that after the year 2010, there was a decrease in the number of reported cases. This progressive reduction in the number of cases of poisoning after the year 2010 may be related to health measures and more efficient hygiene, adequate food processing, as well as more active surveillance of governmental institutions and the implementation of prevention programs and educational activities by educational institutions and other public organs. It must taken into account that in 2013, data were collected only until September.

When assessing the age ranges involved in poisoning events, the age group of 0-9 years was prevalent in cases of poisoning. This profile was also observed in the city of Juiz de Fora, Brazil, in the period 2000-2004^15^ and in the state of Piauí, Brazil, in the period 2007-2012.^16^ A similar representativeness of poisoning cases has been found in children aged 0-9 years and in adolescents aged 10-17 years, in the period of January 2006 to December 2011 in a pediatric emergency service in Hospital de Cova Beira, Portugal.^17^

The age group of 0-4 years is also noteworthy, in which there were 54 recorded cases (43.2%) of poisoning. According to the literature, children younger than 5 years of age have accidents, especially from putting substances or colored objects to the mouth,^4^ in addition to the fact that at this stage, the child begins to walk, becomes more agile, and curiously seeks new things; such peculiarities of child growth and development can cause these accidents.^18^^,^ 19

Over 70% of cases of poisoning in the age group of 0-4 years have been reported in other studies, between the years 2008 to 2010.^4^^,^ 5 ^,^ 18

As for gender, the female gender showed a higher incidence of poisonings, with a total of 69 cases in the age group of 0-19 years.

These data were similar to those described in a study carried out in Minas Gerais in the periods of 2001-2005 and 2005-2007, in which 57% of cases occurred in females aged 0 and 55 years.^20^ When analyzing the profile of poisonings in children and adolescents in a hospital emergency department, Marques also observed that females accounted for 70% of cases of poisoning.^17^ The higher incidence of poisonings in women found in this study was due to a more significant number in the age range of 15-19 years, and medications were the substances most often involved in poisonings. The high prevalence of drug-related toxic events in females has also been reported by other authors.^21^^,^ 22 However, these findings are controversial, because there are studies that show a predominance of reported poisonings in males.^4^^,^ 15 ^,^ 23

When observing the manifestation of toxic effects after consumption of food or drink, the present study considered the presence of poisoning caused by these products and thus, it was observed that these were the most frequent toxic agents (38.4%). Lower frequencies were found in Ceará, Brazil, between 2007 and 2008 (33% of cases)^24^ and in Bahia, Brazil, between 2008 and 2011, where only 12% of cases of poisonings were caused by food or drink.^23^ Considering that the collected data are obtained from epidemiological records of SINAN, it was not possible to differentiate the infectious processes involved in food poisoning.

Reports of food poisoning had a high frequency in this study; however, this type of poisoning has been little reported in studies of exogenous poisoning in children and adolescents. These findings may be related to the fact that, in Barra do Garças, the mean temperature is high throughout the year (with a minimum mean temperature of 12ºC in July and maximum mean temperature of 34ºC),^25^ favoring microbial contamination and food spoilage. The need for laboratory diagnosis should be emphasized in all cases with a history of suspected food poisoning, as the signs and symptoms of such cases are nonspecific and common to other pictures of intoxications caused by other agents.^26^

Medications were the second most common triggering agents of recorded poisoning cases. A similar result was found in Bahia, Brazil, with 22.2%,^23^ but frequencies higher than 50% have also reported.^15^^,^ 27 The reason for a high proportion of medication poisoning is probably related to its easy availability in households^26^ and the extensive variety of existing drugs in Brazil. This favors the emergence of problems related to these products, which constitutes a challenge to public health in both developing and developed countries.^28^

As for the main circumstance of occurrence, i.e., accidental ingestion, similar results are observed in the literature.^4^^,^ 23 These accidental poisonings can be justified at age 1-4 years by the curiosity and agility exhibited by children,^19^ whereas, in age range 5-10 years, they are caused by exposure to environmental hazards and risks, as well as living in remote areas, exposing children to environmental dangers, and risks such as accidents with venomous animals, contamination by heavy metals in vacant lots, and playing in improvised soccer fields.^4^^,^ 5 Also in relation to circumstances, attempted suicide in the state of Bahia^23^ accounted for 25% of cases, similar to the data in this study. Suicide attempts have been increasing worldwide, especially among adolescents and young adults.^29^^,^ 30 The factors that contribute to this situation are current conflicts with the sexual/affective partner, conflicts in the family or at school, mental illness, and disrupted family structures.^31^

The oral route was the most prevalent in poisoning events. Similar results were observed in the period 2000-2004 in the state of Minas Gerais, Brazil, with a percentage of 63% of 86 analyzed poisonings^15^ and, between 2004 and 2010, representing 68% and 74% of the cases, respectively.^4^^,^ 30 It was observed that previous studies showed that medications and oral exposure were triggering factors in poisoning events because they are easily administered, which constitutes the main route of entry of the toxic agent. Regarding seasonality, there have been reports that, in most years, the highest number of poisonings occurred during the warm months.^17^ In this study, the cases were distributed throughout the year; however, the previously described climate characteristics of this municipality should be emphasized. 

Moreover, the time between ingestion and treatment is very important to evaluate the involved risk and to define the best treatment choice. The effectiveness of the initial approach to reduce exposure to toxic agents, either by gastrointestinal decontamination or increased removal of these substances, will also depend on the time elapsed between the occurrence of poisoning and the treatment used. Thus, notification failure regarding the time between ingestion of the toxic products and the notification of the event, observed in a significant percentage of the studied cases, prevents planning and training for proper care of the cases in the municipality.

Considering that poisoning is a process that occurs after exposure to a toxic agent, it is necessary for poisoning cases to be confirmed, which can be attained by laboratory assessment, and clinical and epidemiological analysis. The results found in Barra do Garças, with confirmation of 80% of the reported cases, were similar to that recorded in Sobral, Ceará, Brazil, in the years 2007 and 2008, with 85% of notifications confirmed as poisoning events.^24^

Exogenous poisoning events constitute a common concern, as in addition to the numerous potentially dangerous substances to which humans are exposed, new chemical molecules are constantly discovered and enter use, either domestically or professionally, which can cause harmful effects to humans, especially in children and adolescents. Therefore, studies on the poisoning profile in children and adolescents in Brazilian cities, especially those with a small number of inhabitants, are of utmost importance, as they show different toxicological events, which need to be taken into account. Considering the cases reported in this study, the importance and the need for investment in health control measures is emphasized, as well as educational measures to prevent the occurrence of these poisoning events.
