# Supplementary material for: Epidemiological profile of exogenous poisoning in children and adolescents from a municipality in the state of Mato Grosso
Source: Rev Paul Pediatr. 2014 Dec;32(4):299–305. doi: 10.1016/j.rpped.2014.06.002 (PMC4311782; doi:10.1016/j.rpped.2014.06.002)
Supplement: Translation

# Translation: Perfil epidemiológico das intoxicações exógenas em crianças e adolescentes em município do Mato Grosso☆

**Authors:** Felipe Ferreira S. Oliveira, Eliane Aparecida Suchara

**Affiliations:** Universidade Federal de Mato Grosso (UFMT), Barra do Garças, MT, Brasil

**Keywords:** Intoxicação, Criança, Adolescente

## Abstract

**OBJETIVO:**

Caracterizar o perfil epidemiológico das intoxicações exógenas ocorridas em crianças e adolescentes em Barra do Garças, Mato Grosso, no período de janeiro/2008 a setembro/2013.

**MÉTODOS:**

Foi realizado um estudo epidemiológico descritivo transversal e retrospectivo

Os dados foram coletados através do Sistema de Informação de Agravos de Notificação (SINAN) do município e processados no Microsoft Excel e avaliados através do programa estatístico BIOESTAT. As variáveis avaliadas foram: sexo, idade, agente tóxico, local e tempo de atendimento, via de administração, circunstância e classificação final da intoxicação. A faixa etária foi a estabelecida segundo o Instituto Brasileiro de Geografia e Estatística: crianças com idade de 0 a 9 anos e adolescentes de 10 a 19 anos.

**RESULTADOS:**

Foram registrados 125 casos de intoxicações exógenas, sendo 77 em crianças e 48 em adolescentes. Os principais agentes tóxicos responsáveis pelas intoxicações foram alimentos e bebidas (38,4%) e medicamentos (24,0%). As faixas etárias mais acometidas por intoxicações foram: 0-4 anos (43,2%) e 10-14 anos (19,7%). Em relação às circunstâncias, as intoxicações ocorreram por tentativa de suicídio (16,8%) e acidental (23,2%), respectivamente em adolescentes e crianças. O estudo demonstrou maior frequencia de intoxicações no sexo feminino.

**CONCLUSÕES:**

Conclui-se que as intoxicações exógenas ocorreram predominantemente em crianças até 4 anos por meio do consumo de alimentos ou bebidas e de forma acidental. Assim, é necessária a adoção de medidas educativas de prevenção para os familiares e cuidadores de crianças.

## Introdução

Intoxicação é definida como uma manifestação clínica dos efeitos nocivos produzidos em um organismo vivo como resultado da sua interação com alguma substância química (exógena).^1^ Todos os anos são registrados no Brasil milhares de casos de intoxicação, seja pela ingestão de alimentos contaminados, medicamentos, uso de agrotóxicos, produtos de limpeza doméstica, de uso veterinário e outras substâncias químicas.^2^

As intoxicações, principalmente as não intencionais, constituem uma das principais causas de atendimento de emergência pediátrica.^3^ Quanto aos principais elementos associados aos riscos de uma intoxicação infantil, pode-se relatar que estes estão relacionados à via de exposição oral e ao consumo de medicamentos, sendo que a maioria acontece na própria residência, onde a presença dos pais não impede a sua ocorrência.^4^^,^ 5 Também, esses envenenamentos são mais frequentes em famílias com número superior a três crianças e em pais de baixo nível educacional e de baixa renda.^6^

Observa-se que as intoxicações acidentais, típicas da faixa pediátrica, diminuem no decorrer do desenvolvimento, com a evolução emocional e cognitiva, a qual se torna um possível escape para os problemas em adolescentes, devido ao amadurecimento da concepção de morte.^7^ Em adolescentes, há a necessidade de estudos detalhados, com maior enfoque sobre as circunstâncias e a intenção dessas intoxicações.^8^ Portanto, uma anamnese correta dessas crianças e adolescentes deve ser praticada para que haja um tratamento adequado a esses pacientes, porém os pediatras e os médicos devem estar cientes das peculiaridades dos agentes tóxicos que causam a intoxicação de acordo com a idade e tempo.^9^

No Brasil, os dados sobre intoxicações são disponibilizados nas publicações anuais do Sistema Nacional de Informações Tóxico-Farmacológica (SINITOX), que compila as informações dos 36 Centros de Controle de Intoxicações (CCIs) localizados em 19 estados e no Distrito Federal.^3^ Dados do SINITOX de 2010 relataram 29.554 registros casos registrados de intoxicações em crianças de 0 a 9 anos e 13.087 casos de intoxicações em adolescentes de 10 a 19 anos, com maior prevalência de medicamentos como agente tóxico na faixa etária de 1 a 4 anos.^10^ Na região Centro-Oeste, no ano de 2010, o SINITOX relatou 3.533 intoxicações em crianças de 0 a 9 anos e 1.371 casos em adolescentes de 10 a 19 anos, sendo os medicamentos os mais prevalentes nas intoxicações, principalmente na idade de 1 a 4 anos.^11^ Em relação ao sexo, o feminino, apresentou um maior número de casos, sendo os medicamentos os principais agente causador dessas intoxicações.^12^ Quanto à circunstância, os acidentes individuais prevaleceram, com um total de 6.099 casos e, em segundo lugar, a tentativa de suicídio, com 2.166 casos.^13^

Apesar da existência de dados epidemiológicos sobre intoxicações em nível nacional e regional, a realidade em municípios de pequeno porte ainda é desconhecida. Assim, considerando a vulnerabilidade das crianças à intoxicações, a exposição de jovens a diferentes substâncias que podem ser tóxicas, aliado ao fato que existem poucas informações na literatura sobre a caracterização das intoxicações nessa faixa etária em municípios do interior do Estado do Mato Grosso, este artigo teve como objetivo descrever o perfil das intoxicações exógenas em crianças e adolescentes do município de Barra do Garças, Mato Grosso (MT), no período de janeiro de 2008 a setembro de 2013. 

## Método

O presente trabalho consiste em um estudo epidemiológico transversal, retrospectivo e descritivo, referente a intoxicações exógenas em crianças e adolescentes no município de Barra do Garças (MT) no período de 2008 a 2013. O município está localizado no centro geodésico do Brasil, com latitude 15°53'24" S e longitude 52°15'24" W; encravada aos pés da Serra Azul, um braço da Serra do Roncador, banhada pelos Rios Araguaia e Garças e segundo o Instituto Brasileiro de Geografia e Estatística (IBGE) apresentava uma população estimada de 57.791 pessoas no ano de 2013**.**

A identificação e a caracterização dos dados coletados para o estudo foram estabelecidos de acordo com o Sistema de Informação de Agravos de Notificação (SINAN), por meio de uma ficha de notificação de intoxicações exógenas distribuída pelo Ministério da Saúde. Foram analisados todos os casos de intoxicação exógena notificados, independente se a intoxicação era suspeita ou confirmada. O período avaliado foi de janeiro de 2008 a setembro de 2013, com um total de 125 fichas. As variáveis avaliadas foram: sexo, idade, agente tóxico, local e tempo de atendimento, via de administração, circunstância e classificação final da intoxicação. Quanto à classificação das faixas etárias estudadas, estas foram definidas de acordo com o IBGE: crianças de 0 a 9 anos e adolescentes de 10 a 19 anos. 

Inicialmente realizou-se uma análise descritiva através do software Microsoft Excel 2010 e posteriormente os dados foram avaliados através do programa estatístico BIOESTAT 5.0. Foi verificada a normalidade dos dados através do teste Shapiro Wilk e, posteriormente, foram aplicados os testes não paramétricos de Kruskal-Wallis e Kolmogorov-Smirnov. O nível de confiança adotado em todas as análises foi de 95%. Quanto ao coeficiente de incidencia, este foi calculado segundo o total de casos registrados de intoxicação no período estudado pela população do mesmo local e período multiplicado por 1.000. Os dados populacionais utilizados foram baseados na população estimada divulgada pelo IBGE. 

Em observância a Resolução 466/2012 do Conselho Nacional de Saúde, os pesquisadores submeteram a presente pesquisa ao Comitê de Ética em Pesquisa da Universidade Federal de Mato Grosso, o qual foi registrado e aprovado sob o número do CAAE (Certificado de Apresentação para Apreciação Ética): 24213113.3.0000.5587. 

## Resultados

Na tabela 1 está apresentada a distribuição da freqüência relativa (%) dos casos de intoxicações em crianças de 0 a 9 anos e adolescentes de 10 a 19 anos, no período de 2008 a 2013, no município de Barra do Garças, MT. Os resultados demonstram a ocorrência de 77 casos de intoxicações em crianças e 48 em adolescentes, sendo que os coeficientes de incidências, no período estudado, variaram entre 0,05 e 0,64 por 1.000 habitantes para o grupo das crianças e de 0,03 e 0,27 por 1.000 habitantes para os adolescentes. 

**Tabela 1:** Distribuição de casos de intoxicações em crianças e adolescentes por ano estudado, no município de Barra do Garças, MT

A distribuição dos casos de intoxicação segundo a faixa etária estratificada de crianças e adolescentes por gênero está apresentada na tabela 2. Observa-se que mais de 40% do total de episódios de intoxicações ocorreram em crianças na faixa etária de 0 a 4 anos. Quanto ao sexo, na faixa etária de 0 a 14 anos, verificou-se uma maior frequência de intoxicações no sexo masculino. Para a faixa etária de 15 a 19 anos, as mulheres estavam envolvidas na maioria dos casos notificados. Observou-se uma variação significativa no número de intoxicações registradas quando foi avaliado o número de casos de intoxicações ao longo dos anos estudados para a faixa etária de 0 a 4 anos (*p<*0,05). 

**Tabela 2:** Distribuição dos casos de intoxicação segundo a faixa etária e gênero

As intoxicações por faixa etária segundo o agente tóxico, circunstância, via de administração e classificação final estão representadas na tabela 3. Quando avaliados os casos de intoxicação segundo a faixa etária e agente tóxico exposto, destacou-se a faixa etária entre 0 a 4 anos, com 13 casos de intoxicação por medicamento e 16 casos por alimento ou bebida. Quanto ao agente tóxico envolvido, alimento e bebida foram predominantes nas intoxicações em crianças e medicamentos nos adolescentes. Observou-se também, que entre os diferentes tipos de agentes tóxicos encontrados, existiu diferença significativa no número de casos de intoxicações registradas ao longo dos anos estudados (*p<*0,05). Quanto à circustância, o maior número de casos registrados foi de intoxicações acidentais, sendo que aproximadamente 80% destas foram notificadas em crianças de 0 a 4 anos. Diferentemente, nos casos de ingestão de alimento ou bebida aproximadamente 60% da intoxicações acometeram a faixa etária de 5 a 14 anos. Quando avaliado o número de intoxicações registradas em cada ano e comparado com as circunstancias de intoxicações relatadas, observou-se diferença significativa. Portanto, o número de casos de intoxicações variou entre as circunstâncias relatadas (*p<*0,05) ao longo dos anos estudados. 

**Tabela 3:** Representação dos casos de intoxicações por faixa etária (anos) extratificada segundo o agente tóxico, circunstância, via de administração e classificação final

A via de administração que ocorreu o maior número de intoxicação foi a digestiva, com 92 casos, e o local de atendimento predominante foi o hospitalar (n=64) e o ambulatorial (n=49). Atendimentos a crianças de 0 a 4 anos aconteceram no hospital e no ambulatório respectivamente em 23 (36%) e 26 (53%) casos

Quanto ao tempo de atendimento dos casos de intoxicação, as crianças de 0 a 4 anos e os adolescentes de 15 a 19 anos totalizaram 36 intoxicações atendidas em até uma hora. Contudo, o que prevaleceu foram notificações nas quais essa informação foi ignorada. Ao ser avaliada a distribuição mensal do número de intoxicações, observou-se que os meses de março, junho, julho, agosto e novembro apresentaram 22, 16, 20, 17 e 13 casos de intoxicações, respectivamente. Ao avaliar a classificação final do caso, do total de 125 casos registrados, 81 destes foram confirmados.

## Discussão

No período estudado, foi observada uma incidência maior de intoxicações em crianças e adolescentes no ano de 2010 e esses valores não estavam relacionados a algum evento de intoxicação coletiva, pois os casos tiveram uma distribuição constante durante todos os meses deste ano. Portanto, não foi identificado um surto de intoxicação. Verificou-se também que, após o ano de 2010, ocorreu uma diminuição no número de casos registrados. Tal redução progressiva no número de casos de intoxicações após o ano de 2010 pode estar relacionada a medidas sanitárias e de higiene mais eficientes, ao processamento adequado dos alimentos, a uma fiscalização mais ativa de instituições governamentais e à realização de programas de prevenção e ações educativas por instituições de ensino e outros órgãos públicos. Deve-se ponderar que em 2013, os dados foram coletados somente até o mês de setembro.

Quando avaliadas as faixa etárias envolvidas nos eventos toxicológicos, os grupos de 0 a 9 anos foram as prevalentes em casos de intoxicações. Esse perfil também foi observado no município de Juiz de Fora, no período de 2000 a 2004^14^ e no Piauí no período de 2007 a 2012.^15^ Representatividade semelhante de casos de intoxicações foi encontrado em crianças de 0 a 9 anos e em adolescentes de 10 a 17 anos, no período de e janeiro de 2006 até dezembro de 2011, em um serviço de urgência pediátrica do centro hospitalar de Cova Beira, Portugal.^16^ Vale também destacar a faixa etária de 0 a 4 anos, na qual foram registrados 54 casos (43,2%) de intoxicações. De acordo com a literatura, crianças menores de 5 anos de idade sofrem acidentes, principalmente por levarem substâncias ou objetos coloridos à boca^4^,além do fato de que, nesta fase, a criança começa a andar, ganha agilidade e procura pelo novo com curiosidade, Tais peculiaridades do crescimento e desenvolvimento infantil podem causar esses acidentes.^17^^,^ 18 Valores superiores a 70% de casos de intoxicações na faixa etária de 0 a 4 anos foram relatados em outros estudos, entre os anos de 2008 a 2010.^4^^,^ 5 ^,^ 17

Quanto ao sexo, o feminino apresentou as maiores ocorrências de intoxicações, com um total de 69 casos, na faixa etária de 0 a 19 anos. Estes dados foram semelhantes aos descritos em estudo realizado no interior de Minas Gerais, no período de 2001-2005 e 2005-2007, no qual 57% dos casos ocorreram em mulheres entre zero e 55 anos.^19^ Ao analisar o perfil das intoxicações em crianças e jovens em um Serviço de Urgência Hospitalar, Marques observou também que o gênero feminino foi responsável por 70% dos casos de intoxicações.^16^ A maior ocorrência de intoxicações em mulheres encontrada nesse estudo deve-se a um número mais expressivo na faixa de 15 e 19 anos, sendo os medicamentos os mais envolvidos. A alta prevalência de eventos toxicológicos relacionados a medicamentoso no sexo feminino também é relatada por outros autores.^20^^,^ 21 No entanto, esses achados são controversos, pois também são encontrados estudos nos quais há predominância de notificações de intoxicações em homens.^4^^,^ 14 ^,^ 22

Ao se observar a manifestação de efeitos tóxicos após o consumo de alimentos ou bebidas, considerou-se a presença de intoxicação provocada por estes produtos e, assim, observou-se serem estes os agentes tóxicos de maior ocorrência (38,4%). Menores frequências foram encontradas no Ceará entre 2007 e 2008 (33% das ocorrências)^23^ e na Bahia, entre 2008 e 2011, onde apenas 12% dos casos de intoxicações foram ocasionados por alimento ou bebida.^22^ Considerando que os dados coletados são obtidos por ficha epidemiológica do SINAN, não foi possível diferenciar os processos infecciosos envolvidos nas intoxicações alimentares. Os relatos de intoxicações alimentares tiveram uma frequência elevada nesse estudo, no entanto este tipo de intoxicação tem sido pouco relatado em estudos de intoxicação exógena em crianças e adolescentes. Esses resultados podem estar associados ao fato de que, em Barra do Garças, a temperatura média é alta durante o ano todo, apresentando temperatura média mínima de 12ºC em julho e máxima média de 34ºC,^24^ favorecendo a contaminação microbiana e a degradação dos alimentos. Deve-se ressaltar a necessidade de que o diagnóstico laboratorial seja realizado em todos os casos com história de suspeita de intoxicação alimentar, uma vez que os sinais e sintomas deste tipo de intoxicação são inespecíficos e comuns a outros quadros de intoxicações causadas por outros agentes.^25^

O medicamento foi o segundo agente desencadeante de casos de intoxicações registradas. Resultado similar foi encontrado na Bahia, com valores de 22,2%,^22^ mas frequências superiores a 50% também são relatadas.^14^^,^ 26 A razão para uma alta proporção de intoxicação por medicamentos, provavelmente está relacionada à sua fácil disponibilidade em domicílios^26^ e à extensa variedade de medicamentos existentes no Brasil. Isso favorece o surgimento de problemas relacionados a estes produtos, que representam um desafio à saúde pública tanto em países em desenvolvimento como nos desenvolvidos.^27^

Quanto à principal circunstância ocorrida, a acidental, resultados semelhantes são observados na literatura.^4^^,^ 22 Estas intoxicações causadas por acidentes podem ser justificadas na idade de 1 a 4 anos devido à curiosidade e a agilidade da criança,^18^ enquanto que, na faixa etária de 5 a 10 anos, por exposição a perigos e riscos ambientais e devido ao afastamento de suas residências, expondo-se a perigos e riscos ambientais, como os acidentes por animais peçonhentos, contaminações por metais em terrenos baldios e em campos improvisados de futebol.^4^^,^ 5 Ainda em relação à circunstância, a tentativa de suicídio, no Estado da Bahia^22^representou 25% dos casos, apresentando similiaridade aos dados deste trabalho. As tentativas de suicídio estão aumentando em todo mundo, principalmente nos adolescentes e adultos jovens.^28^^,^ 29 Os fatores que contribuem para essa situação estão os conflitos atuais com o parceiro afetivo, no seio da família ou na escola, doença mental e estruturas familiares perturbadas.^30^

A via oral foi a via de exposição mais prevalente para as intoxicações. Resultado semelhante foi observado no período de 2000 a 2004 no Estado de Minas Gerais, com porcentagem de 63% das 86 intoxicações analisadas^14^ e, no período de 2004 e 2010, com valores de 68% e 74% dos casos analisados.^4^^,^ 29 Observou-se que os estudos anteriores apresentaram o medicamento e a via de exposição oral como desencadeantes para intoxicações pela maior facilidade de administração, que constitui a principal via de introdução do agente tóxico. Em relação à sazonalidade, há relatos que, na maioria dos anos, os maiores números de intoxicações ocorreram durante os meses quentes.^16^ Neste estudo os casos foram distribuídos ao longo do ano, no entanto deve-se ressaltar as características climáticas já destacadas deste município. 

Além disso, o tempo entre a ingestão e o atendimento é muito importante para a avaliação do risco envolvido e na definição de qual a melhor escolha terapêutica. A eficácia da escolha de abordagem inicial para diminuir a exposição aos agentes tóxicos, seja por meio de descontaminação gastrintestinal ou aumento da eliminação desses compostos, irá depender também do tempo entre a ocorrencia da intoxicação e o tratamento aplicado. Assim, a não notificação do tempo entre a ingestão do produto tóxico e a notificação do evento, observada em percentual significativo de casos do estudo, impede um planejamento e treinamento para o cuidado adequado dos casos no município.

Considerando que a intoxicação é um processo que ocorre após a exposição a um agente tóxico, surge a necessidadede que os casos de intoxicação sejam confirmados, o que pode ser realizado por meio de exames laboratoriais, exames clínicos e inquériros epidemiológicos. Os resultados encontrados em Barra do Garças, com confirmação de 80% dos casos notificados, foram semelhantes ao registrado no município de Sobral, Ceará, nos anos de 2007 e 2008, com 85% das notificações confirmadas como intoxicações.^23^

As intoxicações exógenas são preocupações cotidianas, pois além das inúmeras substâncias potencialmente perigosas a que o homem está exposto, novas moléculas químicas são descobertas constantemente e passam a ser utilizadas, seja para uso doméstico ou profissional. Estas podem causar efeitos deletérios aos seres humanos sobretudo em crianças e adolescentes. Portanto, estudos sobre o perfil das intoxicações em crianças e adolescentes em municípios brasileiros, principalmente aqueles com pequeno número de habitantes, são de suma importância, pois estes apresentam eventos toxicológicos diferentes que precisam ser considerados. Considerando os casos notificados neste estudo, salienta-se a importância e a necessidade de investimento em medidas de controle sanitário, e de educação para prevenir a ocorrência destas intoxicações.
